# Impact of Time to Castration Resistance on Cytoreductive Radiotherapy in Metastatic Castration-Resistant Prostate Cancer

**DOI:** 10.3389/fonc.2020.606133

**Published:** 2020-12-04

**Authors:** Lixin Mai, Zitong Zhang, Yonghong Li, Ruiqi Liu, Jibin Li, Sijuan Huang, Maosheng Lin, Boji Liu, Wufei Cao, Jianhua Wu, Mengzhong Liu, Fangjian Zhou, Yang Liu, Liru He

**Affiliations:** ^1^ Department of Radiation Oncology, Sun Yat-Sen University Cancer Center, State Key Laboratory of Oncology in South China, Collaborative Innovation Center for Cancer Medicine, Guangzhou, China; ^2^ Department of Urology, Sun Yat-Sen University Cancer Center, State Key Laboratory of Oncology in South China, Collaborative Innovation Center for Cancer Medicine, Guangzhou, China; ^3^ Department of Clinical Research, Sun Yat-Sen University Cancer Center, State Key Laboratory of Oncology in South China, Collaborative Innovation Center for Cancer Medicine, Guangzhou, China

**Keywords:** prognostic factors, overall survival, cytoreductive radiotherapy, time to castration resistance, castration-resistant, metastatic prostate cancer

## Abstract

**Background:**

The role of local radiotherapy in metastatic castration-resistant prostate cancer (mCRPC) remains undefined. This study aimed to identify the value of local radiotherapy and potential candidates for mCRPC.

**Methods:**

A total of 215 patients with mCRPC treated with or without cytoreductive radiotherapy (CRT) between June 2011 and February 2019 were analyzed. Overall survival (OS) was calculated from the onset of mCRPC. The receiver-operating characteristic (ROC) curve was used to find the cutoff point for time to castration resistance (TCR).

**Results:**

One-hundred and fifty-five (72.1%) patients received abiraterone after mCRPC, and 54 (25.1%) patients received CRT. The median TCR was 14.9 months. After a median follow-up of 31.7 months, the median OS was 33.3 months. The Eastern Cooperative Oncology Group (ECOG) performance scores 0–1, oligometastases, abiraterone after mCRPC, CRT, and TCR ≥9 months were independent prognostic factors for better OS. Stratified analyses showed improved survival when CRT was applied to patients treated with abiraterone (HR 0.44; 95% CI 0.23–0.83; P = 0.012) and TCR ≥9 months (HR 0.39; 95% CI 0.21–0.74; P = 0.004). The percentage of PSA response after radiotherapy was higher in patients with TCR ≥9 months compared to those with TCR <9 months. No grade 3 or worse adverse events after radiotherapy were reported.

**Conclusions:**

ECOG performance score, oligometastases, abiraterone application, TCR and CRT were independent prognostic factors for OS in patients with mCRPC. Patients with a short duration of response to primary androgen deprivation therapy were less likely to benefit from CRT.

## Introduction

Androgen deprivation therapy (ADT) is the basic treatment for metastatic prostate cancer. Notwithstanding, the use of systemic therapy alone can inevitably result in a lethal state, which is castration resistance. Docetaxel (1–3) and the second-generation androgen-receptor-axis-targeted agents (ARATAs) (4–8) provided some survival benefits against metastatic castration-resistant prostate cancer (mCRPC). However, the median overall survival (OS) was reported to be only 23.7 months even if patients were treated with the aforementioned life-prolonging therapies once mCRPC developed (9).

Efforts continue to identify an optimal prognostic model for mCRPC so as to guide individualized treatment strategies. However, most of the previous studies focused only on the impact of baseline status and systemic treatment (10–12). Little is known about the effect of radiotherapy on the clinical outcome of mCRPC, although radiotherapy is usually applied to patients with mCRPC for cytoreduction or palliation in clinical practice.

Increasing evidence indicates that cytoreductive therapy is associated with survival benefits in many metastatic solid tumors, such as metastatic renal cell carcinoma (13, 14), metastatic colon cancer (15), and metastatic hormone-sensitive prostate cancer (16). Recently, a retrospective study with a small sample size (n = 29) showed that radiotherapy directed at oligo-progressive sites might prolong the duration of disease control in patients with mCRPC treated with ARATAs (17). However, data to evaluate the value of CRT in patients with mCRPC is still lacking.

Hence, this retrospective investigation was conducted to figure out prognostic factors for OS after taking CRT into consideration and also to explore the potential benefits of CRT in specific subgroups of patients with mCRPC.

## Materials and Methods

### Patient Selection and Baseline Evaluation

The medical records of 350 consecutive patients with mCRPC treated with abiraterone and/or docetaxel at Sun Yat-Sen University Cancer Center between June 2011 and February 2019 were retrospectively reviewed. Patients with other malignancies or severe uncontrolled medical conditions and those with missing key clinical data or a follow-up of <3 months were excluded, leaving a total of 215 patients with mCRPC in the analysis. This study was approved by the ethics committee of Sun Yat-Sen University Cancer Center (B2020-074).

Oligometastatic disease was defined as ≤5 metastatic lesions (18, 19). CRPC was defined as biochemical or radiological progression in patients receiving ADT with castrate serum testosterone levels (<50 ng/dL) according to the Prostate Cancer Working Group 2 (PCWG2) criteria (20). Time to castration resistance (TCR) was calculated from the time of ADT initiation until the confirmation of CRPC.

### Treatment Approaches

All patients received lifelong ADT (either orchiectomy or gonadotropin-releasing hormone agonist). Systematic treatment after the diagnosis of mCRPC was at the discretion of the treating clinician according to the National Comprehensive Cancer Network guidelines, patients’ preferences, and availability of drugs. Enzalutamide, radium-223, sipuleucel-T, or cabazitaxel was not available before 2020 in mainland China, leaving the choices among abiraterone, docetaxel, and other secondary hormonal therapies (bicalutamide, flutamide, estramustine, etc.). Radiotherapy was applied for tumor cytoreduction and/or symptom palliation. CRT was defined as radiotherapy against lesions that accounted for ≥50% of the total tumor burden (21) by both the treating doctor and the consulting radiologist. Tumor burden was defined as the sum of the longest unidimensional diameter of target lesions according to RECIST 1.1 criteria (22). Patients treated with CRT were categorized as CRT group, while other patients were categorized as non-CRT (nCRT) group.

All patients were simulated with contrast-enhanced computed tomography with site-specific immobilization. Contouring and dose prescription were based on the recommendations by the Radiation Therapy Oncology Group. For the prostate, the clinical target volume (CTV) included the prostate gland with or without seminal vesicles, and the planning target volume (PTV) was obtained by increasing CTV by 5 mm (3 mm posteriorly). Lymph nodes were not irradiated unless radiographically positive. The prescribed dose for the prostate gland and pelvic lymph nodes was 60–67.5 Gy and 45–60 Gy in 25 fractions, respectively. Distant metastases were usually treated with stereotactic body radiation therapy with the dosage regimen of 18–35 Gy/1–5 fractions. The CTV was equivalent to the gross tumor volume. PTV was defined as the CTV plus a variable margin (maximum of 5 mm). Volumetric intensity-modulated arc therapy was used for planning. Image-guided radiotherapy was performed using daily cone-beam CT.

### Outcome Evaluations

The duration of follow-up was calculated from the time of diagnosis of mCRPC. Generally, patients were regularly followed up every 3 months with a prostate-specific antigen (PSA) assessment, and radiological evaluation was arranged at the discretion of physicians when necessary. Acute and late adverse events were assessed according to the Common Terminology Criteria for Adverse Events Version 4.0.

OS was calculated from the date of the diagnosis of mCRPC to the death of any cause or the last follow-up. Progression-free survival after radiotherapy (PFS-RT) was measured from the beginning of radiotherapy until PSA or radiographic progression. PSA response after radiotherapy was defined as a >50% decline in serum PSA levels from the baseline before radiotherapy, according to PCWG2 (20).

### Statistical Analysis

Data were summarized by frequency for categorical variables and by median with interquartile range (IQR) for continuous variables. TCR was dichotomized by calculating the area under the ROC curve using OS as the gold standard to identify the optimal cutoff time. Categorical data were compared using the chi-square test. Survival rates were estimated using the Kaplan–Meier method and compared with the log-rank test. Multivariate analysis was performed using Cox proportional hazards regression. A P value <0.05 was considered statistically significant. Statistical analyses were performed with SPSS 23.0 software (IBM Corp., NY, USA) and the R statistical software (version 3.6.2).

## Results

### Patient and Disease Characteristics

**Figure 1:**
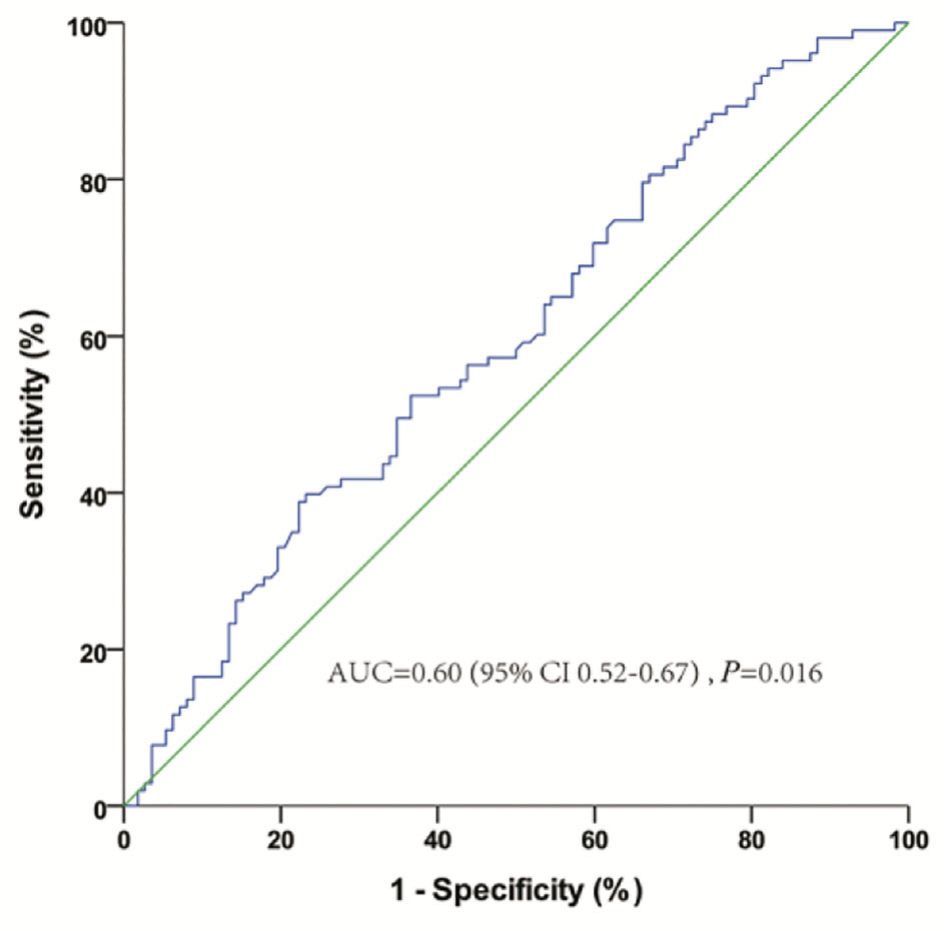
Receiver-operating characteristic curve analysis of the optimal cutoff for the time to castration resistance (TCR) from androgen deprivation therapy.

The baseline characteristics of 215 patients with mCRPC are shown in **Table 1**. The median age was 68 years, and the median baseline PSA was 20.1 ng/ml. Of the 215 patients, most (178, 82.8%) had bone metastases, and 58 (26.7%) had oligometastatic disease. One hundred and fifty-five (72.1%), 98 (45.6%), and 54 (25.1%) patients received abiraterone, docetaxel, and CRT, respectively. Only 3 patients (2 patients in nCRT group, 1 patient in CRT group) receiving PARP inhibitors for BRCA gene mutation. All the patients were treated with ADT or ADT combined with bicalutamide before CRPC, and the median TCR was 14.9 (IQR 7.6–26.8) months. Nine months, which was evaluated as the optimal cutoff value of TCR based on the ROC curve analysis of the association between OS and TCR (**Figure 1**), was used for further survival analysis.

**Table 1:** Baseline characteristics of the 215 patients diagnosed with mCRPC between June 2011 and February 2019.

	No (*N* = 215).	%
Median age (year) (IQR)	68 (62–75)	
ECOG performance score		
0–1	155	72.1
≥2	60	27.9
Median baseline PSA (ng/ml) (IQR)	20.1 (5.4–76.4)	
<20	105	48.8 51.2
≥20	110	
Gleason score		
6–7	58	27 73
8–10	157	
M stage		
M1a	15	7.0
M1b	178	82.8
M1c	22	10.2
Oligometastatic disease		
No	157	73.0
Yes	58	27.0
Abiraterone after mCRPC		
No	60	27.9
Yes	155	72.1
Docetaxel after mCRPC		
No	117	54.4
Yes	98	45.6
CRT after mCRPC		
No	161	74.9
Yes	54	25.1
Median time to CRPC (months) (IQR)	14.9 (7.6–26.8)	
Median F/U (months) (IQR)	31.7 (23.9–50.7)	

CRT, cytoreductive radiotherapy; F/U, follow-up; mCRPC, metastatic castration-resistant prostate cancer; PSA, prostate-specific antigen.

### Analysis of Survival Outcomes and Prognostic Factors

A total of 103 (47.9%) patients died with a median follow-up of 31.7 (IQR 23.9–50.7) months. The median OS and PFS-RT were 33.3 months and 13.1 months for the entire cohort, respectively.

In univariate analyses, TCR, CRT after mCRPC, ECOG performance score, baseline PSA level at mCRPC diagnosis, M stage, oligometastases, abiraterone after mCRPC, and docetaxel after mCRPC were predictive factors for OS (**Table 2**). Multivariate analyses were performed with all variables significant on univariate analyses. TCR [hazard ratio (HR) 0.61; 95% confidence interval (CI) 0.40–0.93; P = 0.021], CRT after mCRPC (HR 0.42; 95% CI 0.24–0.73; P = 0.002), abiraterone after mCRPC (HR 0.43; 95% CI 0.28–0.65; P < 0.001), ECOG performance score (HR 3.36; 95% CI 2.17–5.19; P < 0.001), and oligometastases (HR 0.36; 95% CI 0.20–0.64; P = 0.001) were independent prognostic factors for OS (**Table 2**).

**Table 2:** Univariate and stepwise multivariate Cox hazard analyses of prognostic factors for the overall survival of 215 patients with metastatic prostate cancer.

	Univariate			Multivariate		
Factor	HR	95% CI	*P*	HR	95% CI	*P*
Age (year)						
<68	1		0.159			
≥68	0.76	(0.51–1.12)				
ECOG PS						
<2	1		0.001	1		0.000
≥2	1.97	(1.33–2.91)		3.36	(2.17–5.19)	
PSA (ng/ml)						
<20	1		0.000			0.057
≥20	2.18	(1.45–3.29)				
Gleason score						
6–7	1		0.056			
8–10	1.56	(0.99–2.45)				
M stage						
M1a, M1b	1		0.013			0.277
M1c	1.91	(1.15–3.18)				
Oligometastases						
No	1		0.000	1		0.001
Yes	0.30	(0.17–0.52)		0.36	(0.20–0.64)	
Abiraterone after mCRPC						
No	1		0.001	1		0.000
Yes	0.52	(0.35–0.77)		0.43	(0.28–0.65)	
Docetaxel after mCRPC						
No	1		0.000			0.155
Yes	2.19	(1.46–3.29)				
CRT after mCRPC						
No	1		0.001	1		0.002
Yes	0.43	(0.26–0.72)		0.42	(0.24–0.73)	
TCR (months)						
<9	1		0.001	1		0.021
≥9	0.50	(0.34–0.75)		0.61	(0.40–0.93)	

CRT, cytoreductive radiotherapy; mCRPC, metastatic castration-resistant prostate cancer; PS, performance score; PSA, prostate-specific antigen; TCR, time to castration resistance.

### Stratified Analyses of OS

**Figure 2:**
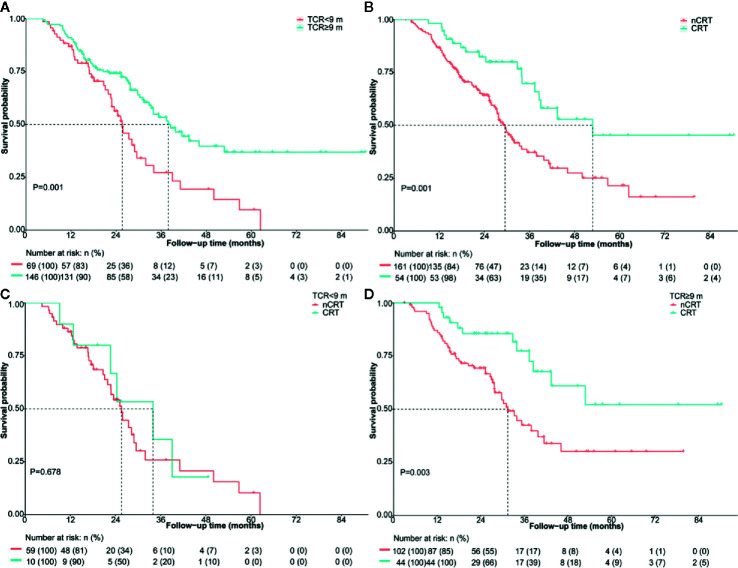
Overall survival of the entire cohort according to **(A)** TCR <9 months vs. TCR ≥9 months **(B)** nCRT vs. CRT for the whole cohort **(C)** nCRT vs. CRT in patients with TCR <9 months **(D)** nCRT vs. CRT in patients with TCR ≥9 months. CRT, cytoreductive radiotherapy; m, months; nCRT, non-cytoreductive radiotherapy; TCR, time to castration resistance.

The median OS of patients with TCR ≥9 months was significantly longer (37.9 vs. 25.6 months, HR: 0.50; 95% CI 0.34–0.75; P = 0.001) (**Figure 2A**). The median OS was 52.8 months and 29.5 months (HR: 0.43; 95% CI 0.26–0.72; P = 0.001) (**Figure 2B**) in patients treated with and without CRT after mCRPC, respectively. The median OS for the patients receiving CRT plus abiraterone, CRT without abiraterone, nCRT plus abiraterone, and nCRT without abiraterone was not reached, 37.4 months, 31.9 months, and 21.9 months, respectively (P < 0.001). The OS of patients with and without CRT stratified by the TCR was compared to explore whether TCR could predict the potential survival benefit of CRT. In the subgroup of patients with TCR <9 months, the OS was similar for patients receiving or not receiving CRT (34.0 months vs. 25.6 months; P = 0.678) (**Figure 2C**). However, the OS was significantly longer when CRT was applied to patients with TCR ≥9 months, (not reached vs 31.4 months; P = 0.003) (**Figure 2D**).

**Figure 3:**
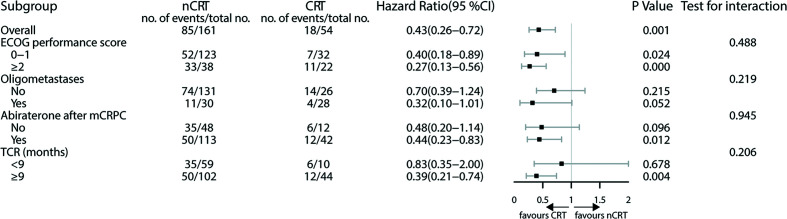
Treatment effect on overall survival within selected baseline categories. CRT, cytoreductive radiotherapy; mCRPC, metastatic castration-resistant prostate cancer; nCRT, non-cytoreductive radiotherapy; TCR, time to castration resistance.

The subgroup analysis showed improved survival when CRT was applied to patients treated with abiraterone (HR 0.44; 95% CI 0.23–0.83; P = 0.012) and TCR ≥9 months (HR 0.39; 95% CI 0.21–0.74; P = 0.004). CRT gave a 68% reduction of death in patients with oligometastatic CRPC, yet the result was not statistically significant (HR 0.32; 95% CI 0.10–1.01; P = 0.052) (**Figure 3**).

### Association Between PSA Response and the Extent of Radiotherapy

Because nCRT group included patients with no RT and RT extent <50% of all disease foci and patients receiving no RT could not be evaluated for PSA response related to radiotherapy, patients were divided into two groups according to the radiotherapy extent: RT <50% group comprising patients receiving radiotherapy targeting less than 50% of all disease foci, and RT ≥50% group comprising patients whose radiotherapy schedules targeted more than or equal to 50% of all disease foci. Overall, 59 (57.3%) of 103 patients receiving radiotherapy had a PSA response. Compared with patients in RT <50% group, patients in the RT ≥50% group had a higher percentage of PSA response (42.9% vs. 70.4%; P = 0.005). Furthermore, when comparing the patients with TCR <9 months, those with TCR ≥9 months obtained a higher percentage of PSA response (41.4% vs. 63.5%; P = 0.041).

### Adverse Events Related to Radiotherapy

Radiotherapy, which was applied to all the 54 patients in CRT group and 49 patients in nCRT group, was generally well tolerated. Acute grade 1–2 genitourinary and gastrointestinal adverse events (AEs) were reported in 16 (29.6%) and 21 (38.9%) patients in the CRT group, respectively. In the nCRT group, for the 49 patients who received RT, acute grade 1–2 genitourinary AEs were observed in 11 (22.4%) patients, and acute grades 1–2 gastrointestinal AEs in 13 (26.5%) patients. Regarding chronic toxicity, only 9 (16.7%) patients in the CRT group had late gastrointestinal AEs, all of which were reported as grade 1. No grade 3 or worse AEs were observed. No treatment interruption or suspension due to radiotherapy was recorded.

## Discussion

Local radiotherapy directed at primary and metastatic sites has been increasingly popular for metastatic hormone-sensitive prostate cancer (mHSPC), but its role in mCRPC remains controversial. The present study provided some clues on the identification of potential candidates for local therapy in the setting of mCRPC. It indicated that CRT was associated with improved survival in mCRPC, especially in patients with good performance status and treated with effective systemic therapies for mCRPC. With regard to potential candidates for local therapy in mCRPC, this study was novel in incorporating prior response duration to ADT into patient selection. Patients with rapid progression to the castration-resistant state (TCR <9 months) were less likely to benefit from CRT.

A growing body of evidence suggests that prostate-directed radiotherapy may provide survival benefits to patients with metastatic prostate cancer. In clinical studies, the prospective randomized controlled trials of HORRAD and STAMPEDE showed survival benefits only in newly diagnosed mHSPC (23, 24). In the case of mCRPC, the value of local therapy is undefined due to the difficulty in identifying the real state of low tumor burden. However, mCRPC represents a heterogeneous cohort, and an intermediate state between localized and widespread disease may exist in certain patients with mCRPC. Some retrospective studies showed that radiotherapy delayed disease progression in patients with oligometastatic CRPC (17, 25–27). Similarly, in the present study, CRT was associated with improved PSA response and OS in mCRPC, indicating that patients with limited metastasis might benefit from local therapy against most or all of metastatic lesions. Current reports on survival benefits of cytoreduction showed varied results. Ongoing trials (NCT 03449719 and NCT04110782) may help examine the survival benefit of local therapy in patients with mCRPC.

It is generally accepted that patient selection is crucial in patients with mCRPC despite different attitudes towards the value of local therapy. Fossati et al (28). reported that the absolute improvement in cancer-specific mortality (CSM)-free survival realized by applying local therapy decreased with an increase in the predicted CSM risk 3 years after diagnosis of metastatic prostate cancer. Local therapy conferred a survival benefit to patients with the CSM risk after 3 years of ≤40%. Our present study also implies that patients with longer life expectancy might benefit from local therapy. On the other hand, next-generation ARAT is an indispensable life-prolonging treatment in the management of mCRPC, and a lower PSA response to next-generation ARAT is believed to be associated with a shorter time to mCRPC. Loriot et al. reported that patients with time to mCRPC of <12 months had a worse PSA response to next-generation ARAT (16% vs. 41%) (29). In the study by Hung et al (30)., the PSA response to next-generation ARAT and the median PFS in patients with rapid, intermediate, and slow progression was 30%, 74%, and 80%, and 3.4, 7.6, and 8.1 months, respectively. Thus, it was speculated that the duration of response to primary ADT predicting response to next-generation ARAT might affect the following OS and might also serve as a screening criterion for local therapy. Our study showed that TCR was not only an independent prognostic factor for OS in mCRPC but also a potential predictive factor for responses and survival outcomes after CRT. Patients with TCR <9 months had similarly poor PSA response and survival outcome irrespective of receiving local therapy, while those who had a relatively long response to ADT showed a significantly higher proportion of response to CRT. Hence, TCR, which is an easily acquired parameter in clinical practice, should be taken into consideration before deciding on local therapy, as patients with longer TCR may represent a cohort with slow progression or good response to subsequent systemic therapy.

Patients in the metastatic castration-resistant state are prone to systemic progression, and a potent systemic control is essential for disease management. The use of abiraterone was found to be an independent prognostic factor, and patients treated with abiraterone in combination with CRT had superior treatment response and survival. Abiraterone was the most commonly used and the most easily accepted agent for mCRPC in China at the time of study. Thus, the results indicated that CRT might be effective only when systemic control could be achieved. The subgroup analyses of the COU-AA-302 and PREVAIL clinical trials revealed that patients with mCRPC might benefit from next-generation ARAT irrespective of tumor burden (6, 31). The increasing novel therapeutic options for mCRPC have made systemic control possible, providing an opportunity for the aggressive local treatment approach. The ongoing FORCE (NCT03556904) trial might shed some light on the value of aggressive local therapy for patients managed by the current standard of care for mCRPC.

This study has some limitations. First, it is a retrospective, single-center study with a limited number of patients. Second, the techniques used for the radiological detection of metastases are not uniform; they comprise traditional imaging and advanced positron emission tomography-computed tomography. Third, although many factors might contribute to the clinical application of CRT, such as patient’s performance status, metastatic burden, patients’ willingness for aggressive local therapy and financial issues, selection bias may exist in our study, since patients receiving CRT might partly represent a cohort whom the physicians considered suitable for local therapy. Fourth, the different timing of radiotherapy and the different sequence of medication makes it difficult to yield a precise indication and optimal timing for local therapy. Hence, the real survival benefit of CRT might be affected by the limitations and should be cautiously interpreted.

## Conclusions

ECOG performance score 0–1, oligometastases, abiraterone application, CRT, and TCR ≥9 months were prognostic factors for improved OS in patients with mCRPC. TCR might be taken into consideration before deciding on local therapy for mCRPC patients, and those with a short TCR were less likely to benefit from CRT. The present study provided some clues on selecting potential candidates for local therapy in mCRPC, yet still needed further studies to identify the precise indications.

## Data Availability Statement

The raw data supporting the conclusions of this article will be made available by the authors, without undue reservation.

## Ethics Statement

The studies involving human participants were reviewed and approved by ethics committee of Sun Yat-Sen University Cancer Center. Written informed consent for participation was not required for this study in accordance with the national legislation and the institutional requirements.

## Author Contributions

All authors contributed to the study conception and design. Data collection and analysis were performed by LM, ZZ, YHL, RL, JL, SH, MSL, BL, WC, and JW. The original draft of the manuscript was written by LM. Review and editing were performed by LH, YL, MZL, and FZ. LH agrees to be accountable for all aspects of the work in ensuring that questions related to the accuracy or integrity of any part of the work are appropriately investigated and resolved. All authors contributed to the article and approved the submitted version.

## Funding

This work was supported by grants from General Program of National Natural Science Foundation of China (No. 81772483).

## Conflict of Interest

The authors declare that the research was conducted in the absence of any commercial or financial relationships that could be construed as a potential conflict of interest.
